# Intra-Season Variations in Workload Parameters in Europe’s Elite Young Soccer Players: A Comparative Pilot Study between Starters and Non-Starters

**DOI:** 10.3390/healthcare9080977

**Published:** 2021-07-31

**Authors:** Alexandre D. Martins, Rafael Oliveira, João P. Brito, Nuno Loureiro, Sérgio M. Querido, Hadi Nobari

**Affiliations:** 1 Institute of Santarém, Sports Science School of Rio Maior–Polytechnic, 2140-413 Rio Maior, Portugal; rafaeloliveira@esdrm.ipsantarem.pt (R.O.); jbrito@esdrm.ipsantarem.pt (J.P.B.); nunoloureiro@esdrm.ipsantarem.pt (N.L.); 2 Life Quality Research Centre, 2140-413 Rio Maior, Portugal; 3 Research Center in Sport Sciences, Health Sciences and Human Development, Quinta de Prados, Edifício Ciências de Desporto, 5001-801 Vila Real, Portugal; 4 Faculty of Human Kinetics, University of Lisbon, 1649-004 Lisbon, Portugal; sergio_querido@sapo.pt; 5 Department of Physical Education and Sports, University of Granada, 18010 Granada, Spain; hadi.nobari1@gmail.com; 6 HEME Research Group, Faculty of Sport Sciences, University of Extremadura, 10003 Cáceres, Spain; 7 Sports Scientist, Sepahan Football Club, Isfahan 81887-78473, Iran; 8 Department of Exercise Physiology, Faculty of Sport Sciences, University of Isfahan, Isfahan 81746-7344, Iran

**Keywords:** internal load, young soccer, ACWR, monotony, performance, monitoring

## Abstract

Background: The main purpose of the current study was to compare the within-season variations of workload, training duration, acute/chronic workload ratio (ACWR), training monotony ™, and training strain (TS) through session rating perceived exertion (s-RPE) between starters and non-starters. Methods: Seventeen under-17 European male soccer players (age, 16.2 ± 0.3 y, height, 1.8 ± 0.1 m; body mass, 66.5 ± 4.0 kg) divided in two groups: nine starters and eight non-starters, were evaluated over 50 weeks throughout the season. Results: In general, there were load variations for all players during the full-season. RPE tended to decrease during in-season and RPE, training duration and s-RPE did not present significant differences between starters and non-starters. TM and TS presented lower values for starters in mesocycle (M) 4 and M11 compared to non-starters. TS presented lower values for starters in M4 and M11 compared to non-starters, while in M10 a higher value was found for starters when compared to non-starters. ACWR showed differences between starters and non-starters in two of the mesocycles. Conclusions: This study showed that some mesocycles provided higher load for non-starters. This information can alert coaches that non-starter players are likely to try too hard in training to demonstrate their abilities, leading to non-functional overreaching, overtraining syndrome, and then poor performance.

## 1. Introduction

The quantification of workload is necessary when determining load imposed to the athletes, their individual response, and adaptation to training programs. All data derived from workload monitoring provides a better understanding when recovery is needed. It also allows to reduce injury risk, illness, overreaching, or overtraining [1,2].

Workload quantification can be characterized as external and internal. External is related to the physical work performed during a training session or match (e.g., distances covered and/or accelerometry-based variables), while internal is related to psychological and physiological variables such as rating of perceived exertion (RPE) and/or heart rate [3,4].

In youth soccer, workload quantification becomes even more relevant because growth, maturation, and puberty also contribute to expose players to non-traumatic injuries [5]. In addition, there are several studies that have demonstrated the association between workload, and physical fitness, fatigue or the risk of injury [6,7].

In soccer athletes, the RPE and session-RPE (s-RPE) are the most used measures to quantify internal workload [2,8]. For instance, Wrigley et al. [9] quantified the weekly workload through RPE in under (U) 14, 16, and 18 soccer players and reported that training sessions after the match presented the highest RPE values.

According to Impellizzeri et al. [2], internal workload is recommended as a primary measure to monitor athletes because it reflects the experienced training from a specific external load that can be different either between or within athletes. Moreover, the limited resources available in youth soccer may also justify the importance to adopt accessible, easy to use, low-cost, and reliable tools.

In addition, there are some metrics that have also been widely suggested in literature as training monotony I [10,11,12], training strain (TS) [10,11,12], and acute: chronic workload ratio (ACWR) [13,14,15]. TM represents the load variability within the week, while TS represents the load variability multiplied by the acute workload [16], which is the accumulated load during a week. Moreover, chronic workload represents the mean load in the past 4 weeks) and consequently makes ACWR, which represents the relationship between acute and chronic workloads [17].

The use of the aforementioned metrics gives information about weekly load variations and provides individual thresholds to minimize the occurrence of injury, which allows researchers and coaches to better adjust training load and take evidence-based decisions [15,18,19]. Despite there being several studies that demonstrate responses to training, whether it is physical fitness, fatigue, or the risk of injury [1,6,7], the literature is still scarce regarding workload quantification in youth training soccer athletes [11], especially when analysing specific characteristic of the players, such as their status (e.g., starters and non-starters).

The focus during the week will shift towards optimally preparing the players for peak performance in the match, rather than on solely improving their physical capacities [20]. Therefore, there will only be one high-intensity training session during the week, as the other conditional training sessions are replaced by competitive matches. However, since the preparation period is also used to form a starting team, there may also be players who are not exposed to the physically challenging demands of these matches every week. Or conversely, non-starting players can train harder to demonstrate their abilities to the coach. It is crucial to study whether non-starter players are likely to train too hard, leading to non-functional overreaching, overtraining syndrome, and then poor performance. This can be to the detriment of the team when it needs non-starter players. Through the full-season, starters and non-starters could reveal different physiological adaptations [21], and recently, new evidence about workload and the player status has been shown in the scientific literature [12,22,23].

In U17 players, the adaptations through the full-season could be determinant for players increasing their participation in competition, as match days usually are the days with higher soccer-specific practice and load [23]. Especially at the youth level, this could be crucial because soccer teams usually participate in three to five training sessions and only one match per week during the in-season [24]. This is a very important aspect in soccer, highlighting the discrepancies in physical loads, which could impair the use of non-players in official matches [22]. Therefore, it is essential to monitor the weekly workload, in order to understand the impact on physical and psychological well-being and the differences in the workload between starters and non-starters [21,22], which could influence the performance in training and matches.

To the best of the author’s knowledge, there is no research on TM, TS, and ACWR, and comparisons between starters and non-starters, through the full-season in youth soccer players, simultaneously. Based on that, the purpose of this study was two-fold: (a) to describe the in-season variations of workload, training duration, TM, TS, and ACWR through s-RPE; and (b) to compare the aforementioned workload parameters between starters and non-starters. It was hypothesized that variations occurred across the season with a tendency of higher values from the pre-season to the end-season. Moreover, it was also hypothesized that starters presented higher values for the workload measures than non-starters.

## 2. Materials and Methods

### 2.1. Participants

The present study included 17 male U17 soccer players belonging to a Portuguese elite team. They were divided into two groups, starters (n = 9, age: 16.2 ± 0.4 years) and non-starters (n = 8, age: 16.2 ± 0.2 years). The inclusion criteria were regular participation in 80% of weekly training sessions. Players had an average of 5.7 ± 2.1 years of soccer training experience.

The exclusion criteria included: (i) players with prolonged injury or a lack of participation in training for at least 2 consecutive weeks; (ii) those who presented the initial physical fitness tests 2 standard deviations below the squad mean (four players were removed based on this criterion); and (iii) goal keepers, due to differences in training activities and workload in training and matches (two players were removed based on this criterion).

The criteria to define starters were assessed week by week to a player’s attendance time in three consecutive matches (≥60 min in each match) while non-starters were considered those who did not achieve this duration [22].

The participants and their parents were informed of the study design as well as the potential risks and benefits of their participation. After being informed and agreeing with the terms of participation, each player and their parent signed an informed and private consent. The study design was approved by the local institutional scientific committee and ethical standards were ensured based on the standards for the experiments conducted in humans as suggested by the Declaration of Helsinki [25].

### 2.2. Design

This study is a descriptive-longitudinal approach and followed a cohort design conducted during a 12-months period from June 2017 to July 2018 (4 weeks during the pre-season and 46 weeks during the in-season). An approach used in previous studies was adapted in which each month corresponded to a mesocycle [26,27,28].

The number of training sessions and the number of competitive matches is presented in Table 1. TL data were collected over a 50-week period of competition where 43 matches and 168 training sessions occurred. Data from rehabilitation or additional training sessions of recuperation were excluded. This study did not influence or alter the training sessions in any way across the full-season, which means that the coach was completely responsible for the training process. A total of 5100 individual training observations were collected during the study. Players performed four training sessions and one match per week during the season [match day minus (MD-), MD-4; MD-3; MD-2, MD and MD plus (+) 2, (MD+2)]. MD+2 is equivalent to MD-5, however, we opted to use MD+2 because this training session was planned according to the previous match. This approach was adapted from previous studies [20,23,26].

### 2.3. Internal Workload Quantification

During training sessions, CR10-point scale [29], adapted by Foster et al. [30] was applied. Thirty minutes after the end of each training session, players rated their RPE value using an app on a tablet. The scores provided by the players were also multiplied by the training duration, to obtain the s-RPE [30,31]. The players were previously familiarized with the scale during the previous two seasons, and all the answers were provided individually to avoid non-valid scores. Through s-RPE, the following variables were calculated: (i) TM (mean of training load during the 7 days of the week divided by the standard deviation of the training load of the 7 days) [10,11,12]; (ii) TS (sum of the training load for all training sessions during a week multiplied by training monotony) [10,11,12]; and (iii) ACWR (dividing the acute workload, 1-week rolling workload data, by the chronic workload, the rolling 4-week average workload data) [13,14,15].

### 2.4. Statistical Analysis

Data were analysed using SPSS version 22.0 (SPSS Inc., Chicago, IL, USA) for Windows statistical software package. Initially, descriptive statistics were used to describe and characterize the sample. Shapiro-Wilk and the Levene tests were used to assumed normality and homogeneity, respectively. Repeated measures ANOVA was used with Tukey’ *b* post hoc once variables obtained normal distribution (Shapiro-Wilk > 0.05), to compare different M and groups (player status). Hedge’s g effect size with 95% confidence interval was also calculated. The Hopkins’ thresholds for effect size statistics were used, as follows: ≤0.2, trivial; >0.2, small; >0.6, moderate; >1.2, large; >2.0, very large; and >4.0, nearly perfect [32]. Results were considered significant with *p* ≤ 0.05.

## 3. Results

### 3.1. Mesocycle Analysis

Table 2 presents descriptive data for training duration, RPE, s-RPE, TM, TS, and ACWR. The results indicate that the highest RPE occurred in M1 (pre-season) and the lowest RPE occurred in M9. The M1 shows significant differences with M2 (*p* = 0.044; g = 1.12 [0.41, 1.87]), M3 (*p* < 0.01; g = 3.63 [2.58, 4.83]), and M9 (*p* < 0.01; g = 3.95 [2.84, 5.23]). M2 shows significant differences with M3 (*p* < 0.01; g = 3.68 [2.62, 4.89]) and M9 (*p* < 0.01; g = 3.64 [2.58, 4.84]). M3 shows significant differences with M4 (*p* = 0.001; g = −1.88 [−2.73, −1.09]), M5 (*p* = 0.032; g = −1.33 [−2.11, −0.61]), M6 (*p* < 0.01; g = −4.56 [−5.98, −3.33]), M7 (*p* < 0.01; g = −3.52 [−4.69, −2.48]), M8 (*p* < 0.01; g = −3.67 [−4.89, −2.61]), and M9 (*p* = 0.001; g = 2.01 [1.21, 2.87]).

The highest training duration occurred in M1 (pre-season) and the lowest in M11. The M2 shows significant differences with M4 (*p* = 0.025; g = 1.89 [1.11, 2.76]), M5 (*p* < 0.01; g = 2.76 [1.85, 3.77]), M6 (*p* < 0.01; g = 4.68 [3.44, 6.14]), M7 (*p* < 0.01; g = 3.59 [2.54, 4.79]), M8 (*p* < 0.01; g = 5.03 [3.72, 6.57]), M9 (*p* = 0.034; g = 1.41 [0.68, 2.19]), M10 (*p* < 0.01; g = 2.64 [1.75, 3.63]), and M11 (*p* = 0.025; g = 1.49 [0.75, 2.29]). M2 shows significant differences with M4 (*p* = 0.002; g = 1.73 [0.96, 2.59]), M5 (*p* < 0.01; g = 2.52 [1.64, 3.49]), M6 (*p* < 0.01; g = 4.32 [3.14, 5.67]), M7 (*p* < 0.01; g = 3.22 [2.24, 4.34]), M8 (*p* < 0.01; g = 4.67 [3.42, 6.12]), M10 (*p* < 0.01; g = 2.39 [1.54, 3.34]), and M11 (*p* = 0.049; g = 1.44 [0.71, 2.23]). M3 shows significant differences with M6 (*p* = 0.007; g = 1.99 [1.19, 2.87]) and M8 (*p* < 0.01; g = 2.31 [1.46, 3.24]). M7 shows significant differences with M8 (*p* = 0.003; g = 1.88 [1.10, 2.74]) and M11 shows significant differences with M12 (*p* = 0.012; g = −0.93 [−1.65, −0.23]).

The s-RPE presents the highest value in M1 and the lowest in M9. The M1 shows significant differences with M6 (*p* = 0.041; g = 1.08 [0.38, 1.82]), M8 (*p* = 0.005; g = 0.99 [0.29, 1.73]), M9 (*p* < 0.01; g = 4.08 [2.95, 5.39]), and M10 (*p* = 0.028; g = 1.38 [0.65, 2.16]). M2 shows significant differences with M9 (*p* < 0.01; g = 3.90 [2.80, 5.17]). M3 shows significant differences with M9 (*p* < 0.01; g = 3.57 [2.53, 4.76]). M4 shows significant differences with M9 (*p* < 0.01; g = 2.39 [1.54, 3.34]). M5 shows significant differences with M9 (*p* = 0.001; g = 2.24 [1.41, 3.16]). M6 shows significant differences with M9 (*p* < 0.01; g = 3.73 [2.66, 4.95]). M7 shows significant differences with M9 (*p* < 0.01; g = 3.43 [2.41, 4.59]). M8 shows significant differences with M9 (*p* < 0.01; g = 3.58 [2.53, 4.77]). M9 shows significant differences with M10 (*p* < 0.01; g = -2.34 [−3.28, −1.49]) and M12 (*p* = 0.035; g = −1.51 [−2.31, −0.77]).

TM presents the highest value in M12 and the lowest in M2. The M1 shows significant differences with M3 (*p* = 0.040; g = −1.39 [−2.18, −0.66]). TS presents the highest value in M12 and the lowest in M9. The M1 shows significant differences with M9 (*p* < 0.01; g = 2.51 [1.64, 3.48]). The M3 shows significant differences with M9 (*p* = 0.023; g = 1.42 [0.68, 2.20]). The M7 shows significant differences with M9 (*p* = 0.043; g = 1.61 [0.85, 2.42]). The M9 shows significant differences with M12 (*p* = 0.021; g = −1.54 [−2.35, −0.79]).

Finally, ACWR presents the highest value in M10 and the lowest in M9. The M1 shows significant differences with M7 (*p* = 0.036; g = −0.18 [−0.86, 0.49]). The M3 shows significant differences with M7 (*p* = 0.044; g = −0.91 [−1.64, −0.22]). The M7 shows significant differences with M9 (*p* = 0.004; g = 1.75 [0.98, 2.58]).

Table 3 shows differences between starters and non-starters during the 12 mesocycles for all variables. Regarding RPE, training duration, and s-RPE, there were no significant differences between starters and non-starters. Match duration differs between starters and non-starters in M3 (*p* = 0.008; g = 1.12 [0.12, 2.20]), M4 (*p* = 0.039; g = 0.08 [−0.86, 1.04]), M6 (*p* = 0.002; g = 1.37 [0.35, 2.51]), M7 (*p* = 0.003; g = 1.25 [0.24, 2.36]), M8 (*p* = 0.005; g = 1.27 [0.26, 2.39]), and M10 (*p* = 0.002; g = 2.25 [1.08, 3.62]). TM differs between starters and non-starters in M4 (*p* = 0.027; g = −1.12 [−2.12, −0.12]) and M11 (*p* = 0.005; g = −2.26 [−3.64, −1.09]). TS presents significant differences between starters and non-starters in M4 (*p* < 0.01; g = −1.83 [−3.08, −0.73]), M10 (*p* = 0.007; g = 1.44 [0.40, 2.59]), and M11 (*p* = 0.007; g = −1.44 [−2.60, −0.412]). Finally, the ACWR shows significant differences between starters and non-starters in M4 (*p* = 0.015; g = −1.26 [−2.37, −0.25]) and M5 (*p* = 0.039; g = 1.04 [0.05, 2.11]).

### 3.2. Microcycle Analysis

**Figure 1:**
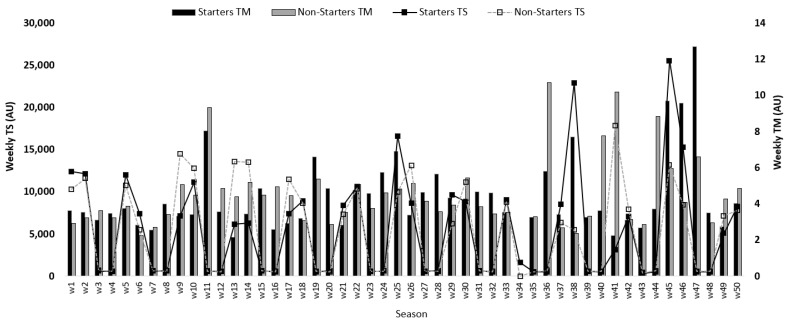
Descriptive statistics of microcycle average for training monotony (TM) and training strain (TS) between starters and non-starters.

Figure 1 shows the average weekly variations of the TM and the TS across the full-season for starter and non-starter players. The highest TS occurred in week 45 for starters and for non-starters occurred in week 41. The lowest TS was verified in week 34 for both starters and non-starters. About the TM, the highest occurred in week 47 for starters and for non-starters occurred in week 36. The lowest TM was verified in week 34 for both starters and non-starters.

**Figure 2:**
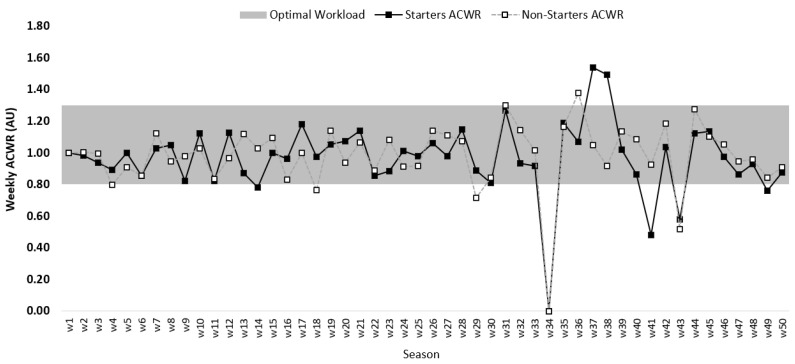
Descriptive statistics of microcycle average for ACWR between starters and non-starters.

Figure 2 shows the average weekly variations regarding the ACWR for the full season for starters and non-starters. In general, the values remained in the optimal zone until week 34. The lowest values for starters and non-starters were recorded at week 34. After this week, the values for starters were higher and remained outside the optimal zone. In the last 5 weeks of the season, they stabilized in the optimal zone for starters and non-starters.

### 3.3. Match-Day-Minus Analyses

**Figure 3:**
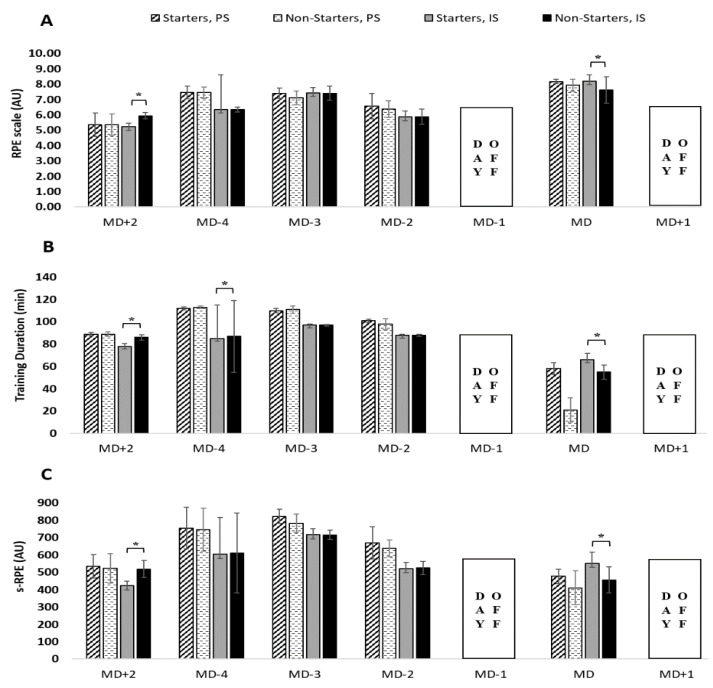
Training load in full-season for RPE (**A**), training duration (**B**), and s-RPE (**C**) with respect to days before a competitive match between player status. Abbreviations: PS—Pre-season; IS—In-season; (*) denotes significant differences between starters with non-starters, all *p* ≤ 0.05.

In the pre-season, RPE of training sessions (Figure 3A) presents the highest and the second highest during MD-4 and MD-3, respectively. During in-season, the highest and the second highest occurred in MD-3 and MD-4, respectively. MD+2 presents the lowest value for both phases. No differences were found between starters and non-starters in the pre-season, while there were significant differences in MD+2 (*p* = 0.001; g = −3.15 [−4.81, −1.78]) and MD (*p* = 0.022; g = 0.83 [−0.13, −1.87]) during in-season.

In the pre-season, the training duration (Figure 3B) was the highest and the second highest in MD-4 and MD-3, respectively. In-season, the highest and the second highest occurred in MD-3 and MD-4, respectively. MD+2 presented the lowest value for both phases. No differences were found between starters and non-starters in the pre-season, while there were significant differences in MD+2 (*p* < 0.01; g = −3.05 [−4.68, −1.71]), MD-4 (*p* = 0.036; g = −0.06 [−1.01, 0.88]), and MD (*p* = 0.005; g = 1.77 [0.69, 3.01]) in-season.

Figure 3C revealed that s-RPE presents the highest and the second highest in MD-3 and MD-4, respectively, while MD+2 presents the lowest values. No differences were found between starters and non-starters in the pre-season, while there were significant differences in MD+2 (*p* < 0.01; g = −2.23 [−3.60, −1.06]) and MD (*p* = 0.011; g = 1.33 [0.31, 2.46]) in-season.

## 4. Discussion

The purposes of the present study were: (a) to characterize the pre-season and in-season workload variation; and (b) to compare the full-season between starters and non-starters of European U17 soccer players. The major finding revealed a meaningful variation in the workload indices for the squad average. The results indicated that RPE tended to decrease and flatten in-season, where in M3 and M9 the lowest values were found. While M9 is easily explained by the lowest volume in training, M3 is possibly explained by a greater focus of the coach in the tactical preparation of the squad. However, the athletic performance improvement is an adaptive process that requires manipulation of the workload imposed on the players; the lower intensity applied in that mesocycle may explain the lower RPE values [33].

Regarding RPE, higher values were found for the full-squad during the pre-season (M1) when compared to other mesocycles, which is in line with several authors [10,11,34]. During this period, a higher workload was achieved to prepare players for the competitive phase. However, most mesocycles have similar RPE values, which cause a tendency to flatten out differences in RPE. Nevertheless, it seems that varying short term workload according to the different developmental ages benefits players’ physical performances [35]. In the present study, the variations that occurred in the microcycles did not have an expression in the total workload of the mesocycle, justifying the reduced variations observed. For instance, Wrigley et al. [9] presented higher RPE values during training sessions than in matches for U14, 16, and 18, and eventually, these variations may have occurred and contributed to the results recorded in the microcycles and mesocycles of the present study. The comparison of RPE between starters and non-starters showed significant differences in MD+2 and MD (Figure 3A). This result is in line with a previous study [36]. In fact, MD+2 is very important for the recovery of the team (namely, the players who were starters) and to prepare players for the next training sessions with higher-intensity (MD-4 and MD-3).

Regarding training duration for the entire-squad, it was higher than the values reported by some authors in elite professional teams [1,22] and in elite U17–19 soccer players [9,21], however, it is similar to the values reported by Brink et al. [37] in elite U19 soccer players. Moreover, as reported by Coutinho et al. [38], the lower training frequencies in the youngest groups were balanced by a higher training stimulus. However, as the focus moved towards the competition rather than the player development, there was a progressive decrement of the individual responses to training as the competition congestion fixture approached [2].

Concerning s-RPE, there were no differences between the starters and non-starters in the pre-season phase (M1). The 6 friendly matches and the 17 training sessions that occurred in M1 may have imposed a greater physical demand on players, allowing a similar workload for all squad players. Also, in M2 and M3 there were no differences between the starters and non-starters for all variables, which can perhaps be explained by a higher turnover of players, since the main 11 starters were not yet well defined in that period of the season. The highest value of s-RPE was presented in M1 and the lowest was presented in M9, which is similar to another study [37]. Furthermore, the highest training duration and s-RPE occurred in M1 for both starters and non-starters, and they were in line with some studies [10,22,34], which means that the exercise training program focused on improving physical conditions through a higher training load during the pre-season. In match-day-minus analyses, regarding s-RPE, it was possible to identify significant differences in MD+2 and MD (Figure 3C). The explanation for this difference was the fact that the coach and his technical team planned the MD+2 with two distinct concerns: the concern for starters was to decrease muscle soreness and mental fatigue, and to restore the hormonal balance, while the concern for non-starters was to increase the physical, tactical, and technical levels.

Regarding TM, the highest value for the full squad was observed in M3 and M12. A previous study [11] reported that weekly TM showed reduced variation over the season (e.g., weekly changes: −8.8–7.5%). In the present study, TM seemed to stabilize during the middle-season, increasing in the last mesocycle, probably because decisive matches were played (seven matches). Similar findings were found for TM during the season (3.17–5.98 AU) [10]. Regarding the comparison of TM between starters and non-starters, only in M4 and M11 were differences found. However, in professional players, Nobari et al. [22] reported that starters presented significantly higher values of TM and TS (calculated through accelerometer variables) across the full season.

The highest value of TS occurred in M12 and it can possibly be justified by the decisive matches and the need to keep the team at the highest competitive level. Our study also observed higher absolute values of TS (7836.65 AU) than those reported in the literature [9,11], but they are in line with those reported by Arazi et al. [6] (6.930–16.800 AU). In addition, M12 presented the largest number of microcycles and matches, which may have generated a greater accumulated fatigue in the players, and consequently higher values of TM and TS. It is likely that reduction in TS in some periods of the season was a deliberate attempt to reduce training volume and to provide adequate recovery to maintain fitness and freshness [22]. The weekly TS showed a “w-shape” and in M10 the highest TS value of the full-season occurred for starters, while non-starters presented the highest value in M11.

Although the values of TM and TS in the present study are higher than those reported in the literature [39], these metrics showed insufficient evidence for an association with injury risk. In fact, only one traumatic and one muscle injury occurred for starters, and only one injury with a different cause from soccer occurred for a non-starter player.

Finally, when analysing ACWR for the full squad, the highest value occurred in M10 and the lowest in M9. Regarding ACWR comparison between starters and non-starters, there were differences in M4 and M5. Possible justifications could be associated with the differences in M3 and M4 for match duration, which may have motivated the attribution of an additional training session to non-starters by the coach, thus influencing the ACWR. Furthermore, ACWR range values were according to the references in the literature [13,14,18] for almost the full-season.

Although the values of TM and TS in the present study are higher than those reported in the literature [10,11,22], Verstappen et al. [39] stated that TM and TS showed insufficient evidence for an association with injury risk, which supports the idea that the values found in the present study could be used as a reference in U17 soccer players. In addition, the effects of training and matches on perceived workload parameters indicated that the amount of training and matches was well tolerated by the players over the investigated season. Thus, at this competitive level, coaches and technical staff should not consider only one marker for injury risk (e.g., TM), but perform a global analysis of all parameters described in this study.

As limitations, this study only presented RPE to quantify workload, and that may have constrained the real training and match quantification of physiological responses. Despite the players being previously familiarized with the scale during the previous two seasons, there was a minor possibility that players would have answered the questionnaire to minimize fatigue effects and thus not reported the actual proper rate. Therefore, further studies may include distance at different intensity zones and accelerometery-based workload data to describe the weekly load variations for starters and non-starters over the season.

In addition, the small sample size, the specific team, their age, and competition does not allow for the generalizability of the results to other scenarios and requires replication studies. Furthermore, for future research, the complete description of training programs can allow a better transfer to other teams. Finally, contextual variables such as match location, results, and opponent quality should be considered in future studies as previously recommended [40,41].

## 5. Conclusions

In general, the findings revealed that the highest values of TM and TS occurred from the middle of the season to the end of the season with higher values presented for non-starters, which is not in line with the literature. In addition, ACWR range values were according to the literature for a full season with small specific exceptions. Also, the present study revealed higher TM and TS values than usual, which led us to conclude that reference values for this age group could be not properly established, and for that reason, future studies are required to confirm the present results. The typical microcycle distribution scheme between every weekday with MD can help coaches and staff team to understand how to distribute the training load according to status, with MD+2 being the most relevant day. Ultimately, this information can alert coaches that non-starter players are likely to practice hard training workload to demonstrate their abilities, leading to non-functional overreaching and overtraining syndrome, and, consequently, poor performance.

## Figures and Tables

**Table 1:** Description of macrocycle of the present study.

Mesocycle (M)	M1	M2	M3	M4	M5	M6	M7	M8	M9	M10	M11	M12
Phases	Pre-season	Early-season			Mid-season					End-season		
Number of weeks	4	4	4	4	4	4	4	4	4	4	4	6
Training sessions (N)	17	16	15	14	14	14	15	15	9	15	12	12
Training duration, total minutes, ST	1676	3170	1316	1118	1235	1227	1204	1190	802	1197	791	1365
Training duration, total minutes, NST	1706	1576	1353	1319	1177	1188	1325	1218	939	1166	1022	1491
Match duration, total minutes, ST	198	194	242	193	223	205	241	183	122	221	0	312
Match duration, total minutes, NST	178	82	130	190	153	105	110	90	46	61	0	153
Number of matches (N)	4 *	4	4	4	4	4	4	3	2	3	0	7

Abbreviations: *, friendly matches; ST, Starters; NST, Non-starters; N, number.

**Table 2:** Training load data during the 12 mesocycles for squad average, Mean ± SD.

Mesocycle (M)	RPE (AU)	Training Duration (min)	s-RPE (AU)	TM (AU)	TS (AU)	ACWR (AU)
M1	7.08 ± 0.57 **^a,b,h^**	96.10 ± 2.2 **^c,d,e,f,g,i,j^**	640.62 ± 56.67 **^d,g,h,i^**	3.35 ± 0.46 **^b^**	6133.19 ± 1053.64 **^h^**	0.95 ± 0.77 **^f^**
M2	6.55 ± 0.32 **^b,h^**	95.40 ± 2.2 **^c,d,e,f,g,i,j^**	594.88 ± 31.13 **^h^**	3.17 ± 0.72	4797.76 ± 1704.91	0.97 ± 0.95
M3	5.4 ± 0.29 **^c,d,e,f,g,h^**	92.30 ± 3.2 **^e,g^**	593.86 ± 50.62 **^h^**	5.22 ± 1.79	5927.38 ± 2697.09 **^h^**	0.96 ± 0.64 **^f^**
M4	6.57 ± 0.81 **^h^**	88.15 ± 5.35	555.31 ± 93.44 **^h^**	3.96 ± 1.43	5132.58 ± 2651.22	0.97 ± 0.11
M5	6.47 ± 1,07 **^h^**	88.10 ± 3.35	540.52 ± 93.27 **^h^**	4.18 ± 1.35	4813.15 ± 2396.26	1.02 ± 0.11
M6	6.9 ± 0.35 **^h^**	87.25 ± 1.40	588.04 ± 36.05 **^h^**	4.36 ± 1.27	4909.88 ± 1705,96	0.98 ± 0.84
M7	6.7 ± 0.42 **^h^**	89.25 ± 1.45 **^g^**	578.06 ± 45.99 **^h^**	4.8 ± 1.52	6355.97 ± 2703.55 **^h^**	1.05 ± 0.05 **^h^**
M8	6.78 ± 0,43 **^h^**	86.4 ± 1.5	588.22 ± 45.66 **^h^**	4.32 ± 1.6	4783.82 ± 2416.69	0.99 ± 0.09
M9	4.01 ± 0.91 **^i,j,k^**	83.2 ± 12.40	333.62 ± 87.09 **^i,k^**	3.45 ± 1.28	2733.05 ± 1547.62 **^k^**	0.85 ± 0.15
M10	6.25 ± 0.99	88.45 ± 3.35	538.98 ± 84.08	4.28 ± 1.47	5834.13 ± 4015.37	1.14 ± 0.29
M11	6.51 ± 1.88	75.3 ± 19.10 **^k^**	503.1 ± 149.79	4.5 ± 2.62	4632.08 ± 3199.87	0.89 ± 0.25
M12	6.42 ± 1.71	88.35 ± 3.50	513.19 ± 139.06	5.98 ± 3.98	7836.65 ± 4293.20	0.94 ± 0.26

Abbreviations: RPE, rating of perceived exertion; s-RPE, session rating of perceived exertion; TM, training monotony; TS, training strain; ACWR, acute: chronic workload ratio; AU, arbitrary units; min, minutes; a, denotes differences with M2; b, denotes differences with M3; c, denotes differences with M4; d, denotes differences with M5; e, denotes differences with M6; f, denotes differences with M7; g, denotes differences with M8; h, denotes differences with M9; i, denotes differences with M10; j, denotes differences with M11; k, denotes differences with M12; *p* ≤ 0.05.

**Table 3:** Differences between starters and non-starters during the 12 mesocycles, Mean ± SD.

Variables	M1	M2	M3	M4	M5	M6	M7	M8	M9	M10	M11	M12
RPE (AU), ST	6.99 ± 0.75	6.59 ± 0.32	5.39 ± 0.19	6.26 ± 0.97	6.58 ± 0.95	6.90 ± 0.35	6.86 ± 0.37	6.77 ± 0.47	3.87 ± 1.27	6.54 ± 0.52	5.86 ± 2.37	6.06 ± 2.33
RPE (AU), NST	7.18 ± 0.31	6.51 ± 0.34	5.41 ± 0.39	6.93 ± 0.39	6.35 ± 1.24	6.92 ± 0.39	6.69 ± 0.48	6.80 ± 0.41	4.17 ± 0.13	5.92 ± 1.31	7.25 ± 0.66	6.82 ± 0.34
TD (min), ST	96.32 ± 2.50	95.81 ± 2.00	92.20 ± 1.30	86.00 ± 6.15	86.50 ± 4.05	88.12 ± 1.45	88.45 ± 1.50	85.45 ± 1.55	82.30 ± 16.10	87.22 ± 2.45	69.55 ± 26.34	75.00 ± 28.14
TD (min), NST	95.00 ± 3.10	95.55 ± 2.45	92.55 ± 4.50	90.10 ± 2.20	90.15 ± 2.55	87.40 ± 2.05	90.15 ± 1.57	87.30 ± 1.40	83.48 ± 7.35	89.51 ± 2.58	81.45 ± 2.55	85.37 ± 2.50
MD (min), ST	69.00 ± 6.05	67.55 ± 17.25	**72.55 ± 6.58**	**66.51 ± 15.03**	68.33 ± 9.58	**74.22 ± 10.26**	**72.00 ± 6.37**	**68.55 ± 8.20**	67.58 ± 28.00	**74.00 ± 7.10**	0.00 ± 0.00	61.44 ± 25.59
MD (min), NST	64.00 ± 9.44	33.38 ± 27.56	**54.50 ± 20.00**	**64.50 ± 6.55**	54.5 ± 15.00	**37.37 ± 33.41**	**50.00 ± 22.05**	**38.00 ± 30.17**	28.55 ± 33.00	**24.00 ± 28.00**	0.00 ± 0.00	47.00 ± 33.44
s-RPE (AU), ST	638.94 ± 68.21	591.94 ± 31.98	594.28 ± 34.46	515.36 ± 114.48	550.48 ± 86.80	589.01 ± 41.70	586.04 ± 38.38	551.91 ± 52.32	317.53 ± 120.09	559.15 ± 53.14	441.26 ± 181.34	483.42 ± 188.39
s-RPE (AU), NST	642.52 ± 44.88	598.18 ± 31.97	593.39 ± 67.08	600.26 ± 24.87	529.33 ± 104.90	586.95 ± 31.30	569.09 ± 54.57	565.33 ± 39.10	351.74 ± 12.16	516.30 ± 108.73	572.68 ± 57.05	546.69 ± 34.90
TM (AU), ST	3.44 ± 0.51	3.28 ± 0.99	4.59 ± 0.94	**3.27 ± 1.16**	4.39 ± 0.93	4.48 ± 1.21	5.13 ± 1.85	4.46 ± 1.82	3.55 ± 1.64	4.50 ± 1.49	**2.94 ± 1.22**	7.04 ± 5.22
TM (AU), NST	3.25 ± 0.42	3.06 ± 0.19	5.93 ± 2.29	**4.75 ± 1.35**	3.95 ± 1.74	4.24 ± 1.40	4.42 ± 1.04	4.16 ± 1.42	3.35 ± 0.81	4.03 ± 1.51	**6.26 ± 2.71**	4.78 ± 1.45
TS (AU), ST	6443.53 ± 1026.87	5172.80 ± 2266.35	4860.10 ± 1496.07	**3396.85 ± 1542.00**	4369.03 ± 1142.45	5054.42 ± 1641.17	6612.66 ± 3311.01	4913.58 ± 1788.51	2936.06 ± 2049.18	**8142.53 ± 4144.94**	**2787.64 ± 1481.85**	9205.20 ± 5405.25
TS (AU), NST	5784.05 ± 1034.05	4375.83 ± 624.07	7128.07 ± 3310.12	**7085.29 ± 2262.19**	5312.79 ± 3330.78	4747.26 ± 1875.31	6067.20 ± 1999.47	4637.84 ± 3106.09	2504.66 ± 750.29	**3237.18 ± 1630.65**	**6707.08 ± 3405.82**	6297.03 ± 1903.08
ACWR (AU), ST	0.95 ± 0.06	0.98 ± 0.07	0.97 ± 0.03	**0.91 ± 0.08**	**1.07 ± 0.07**	0.97 ± 0.08	1.04 ± 0.05	0.97 ± 0.11	0.81 ± 0.20	1.23 ± 0.39	0.81 ± 0.32	0.92 ± 0.36
ACWR (AU), NST	0.95 ± 0.09	0.96 ± 0.12	0.95 ± 0.09	**1.04 ± 0.10**	**0.96 ± 0.12**	0.99 ± 0.09	1.06 ± 0.06	1.00 ± 0.06	0.89 ± 0.06	1.05 ± 0.08	0.98 ± 0.12	0.97 ± 0.06

Significant differences (*p* ≤ 0.05) between starters and non-starters are highlighted in bold. Abbreviations: M, mesocycle; RPE, rating of perceived exertion; TD, training duration; MD, match duration; s-RPE, session rating of perceived exertion; TM, training monotony; TS, training strain; ACWR, acute: chronic workload ratio; AU, arbitrary units; ST, Starters; NST, Non-Starters; min, minutes.

## Data Availability

The datasets used and/or analyzed during the current study are available from the corresponding author on reasonable request.
